# *Capillaria plectropomi* n. sp. (Nematoda: Capillariidae), a new intestinal parasite of the leopard coral grouper *Plectropomus leopardus* (Serranidae) off New Caledonia

**DOI:** 10.1051/parasite/2014076

**Published:** 2014-12-23

**Authors:** František Moravec, Jean-Lou Justine

**Affiliations:** 1 Institute of Parasitology, Biology Centre of the Academy of Sciences of the Czech Republic Branišovská 31 370 05 České Budějovice Czech Republic; 2 ISYEB, Institut Systématique, Évolution, Biodiversité, UMR7205 CNRS, EPHE, MNHN, UPMC, Muséum National d’Histoire Naturelle CP 51, 55 rue Buffon 75231 Paris Cedex 05 France

## Abstract

A new nematode species, *Capillaria plectropomi* n. sp. (Capillariidae), is described from the intestine of the leopard coral grouper *Plectropomus leopardus* (Lacepède) from coral reefs off New Caledonia. The new species, belonging to the subgenus *Neocapillaria* Moravec, 1987, differs from other congeneric species of this subgenus from marine fishes mainly in the length (168–186 μm), shape and structure of the spicule. It is characterized, in the male, by the presence of two well-developed dorsolateral caudal lobes, a pair of lateral papillae, a heavily sclerotized spicule with many rough transverse grooves in the middle part, a spinose spicular sheath, and in the female, by eggs measuring 60–66 × 27 μm without protruding polar plugs. The buccal cavity contains a small finger-shaped stylet. *Capillaria plectropomi* n. sp. is the first known species of this genus parasitizing fishes of the perciform family Serranidae.

## Abstract (fr)

Une nouvelle espèce de nématode, *Capillaria plectropomi* n. sp. (Capillariidae), est décrite de l’intestin de la saumonée *Plectropomus leopardus* (Lacepède) des récifs coralliens au large de la Nouvelle-Calédonie. La nouvelle espèce, appartenant au sous-genre *Neocapillaria* Moravec, 1987, diffère principalement des autres espèces de ce sous-genre qui sont parasites de poissons marins, dans la longueur (168–186 μm), la forme et la structure du spicule. Elle est caractérisée, chez le mâle, par la présence de deux lobes caudaux dorsolatéraux bien développés, une paire de papilles latérales, un spicule fortement sclérifié avec de nombreuses rainures transversales rugueuses dans la partie médiane, une gaine spiculaire épineuse, et chez la femelle par des œufs mesurant 60–66 × 27 μm, sans bouchons polaires saillants. La cavité buccale contient un petit stylet en forme de doigt. *Capillaria plectropomi* n. sp. est la première espèce connue de ce qui est de parasite de poissons perciformes de la famille Serranidae.

## Introduction

The present knowledge of the diversity and biology of capillariid nematodes (Capillariidae) is fragmentary, especially where species parasitizing marine fishes are concerned. In addition, the taxonomy of these pathogenic parasites based on morphological features is rather difficult; therefore, capillariids remain frequently unidentified in faunistic surveys, being reported only as *Capillaria* (s.l.) sp. or Capillariidae gen. sp. [8]. The fauna of these parasites in marine fishes off New Caledonia remains almost unknown, because to date only two nominal species, *Pseudocapillaria echenei* (Parukhin, 1967) and *P*. *novaecaledoniensis* Moravec & Justine, 2010, are known to parasitize *Echeneis naucrates* Linnaeus (Echeneidae) and *Pristipomoides argyrogrammicus* (Valenciennes), respectively, in this region. Seven other morphologically different types of capillariid females, apparently each of them representing a new species, have been reported from *Carangoides oblongus* Cuvier (Carangidae), *Diagramma pictum* (Thunberg) (Haemulidae), *Fistularia commersonii* Rüppel (Fistulariidae), *Naso unicornis* (Forsskål) (Acanthuridae), *Siganus doliatus* (Guérin-Méneville) (Siganidae), *Stegostoma fasciatum* (Hermann) (Stegostomidae) and *Synodus dermatogenys* Fowler (Synodontidae), but, because of the absence of conspecific males, these could not be identified to the genus and species and were designated as Capillariidae gen. spp. [9, 11]. Unidentified capillariids from New Caledonian waters were also reported by Justine et al. [6] from *Plectropomus laevis* (Lacepède) and *P*. *leopardus* (Lacepède) (Serranidae), based on specimens collected from these hosts while studying the parasites of marine fishes off New Caledonia by J.-L. Justine between 2003 and 2011. Closer examination of the specimens from the latter host revealed that they represent a new species, which is described herein.

The leopard coral grouper *Plectropomus leopardus* (maximum body length 120 cm, weight 23.6 kg) is a tropical marine, reef-associated commercial and game fish also used for aquaculture. It is distributed in the Western Pacific from southern Japan to Australia and eastwards to the Caroline Islands, Fiji and Tonga [4].

## Materials and methods

The grouper was caught by line, brought back to the laboratory and immediately examined. The nematodes obtained were washed in physiological saline and were then fixed and preserved in 70% ethanol. For light microscopical examination, the nematodes were cleared with glycerine. Drawings were made with the aid of a Zeiss drawing attachment. Specimens used for scanning electron microscopy (SEM) were postfixed in 1% osmium tetroxide (in phosphate buffer), dehydrated through a graded acetone series, critical-point-dried and sputter-coated with gold; they were examined using a JEOL JSM-7401F scanning electron microscope at an accelerating voltage of 4 kV (GB low mode). All measurements are in micrometres unless otherwise indicated. The fish nomenclature adopted follows FishBase [4].

## *Capillaria plectropomi* n. sp. (Figs. 1, 2)

urn:lsid:zoobank.org:act:326A73A5-540B-4586-9043-4A38B4352BFF

**Figure 1.**
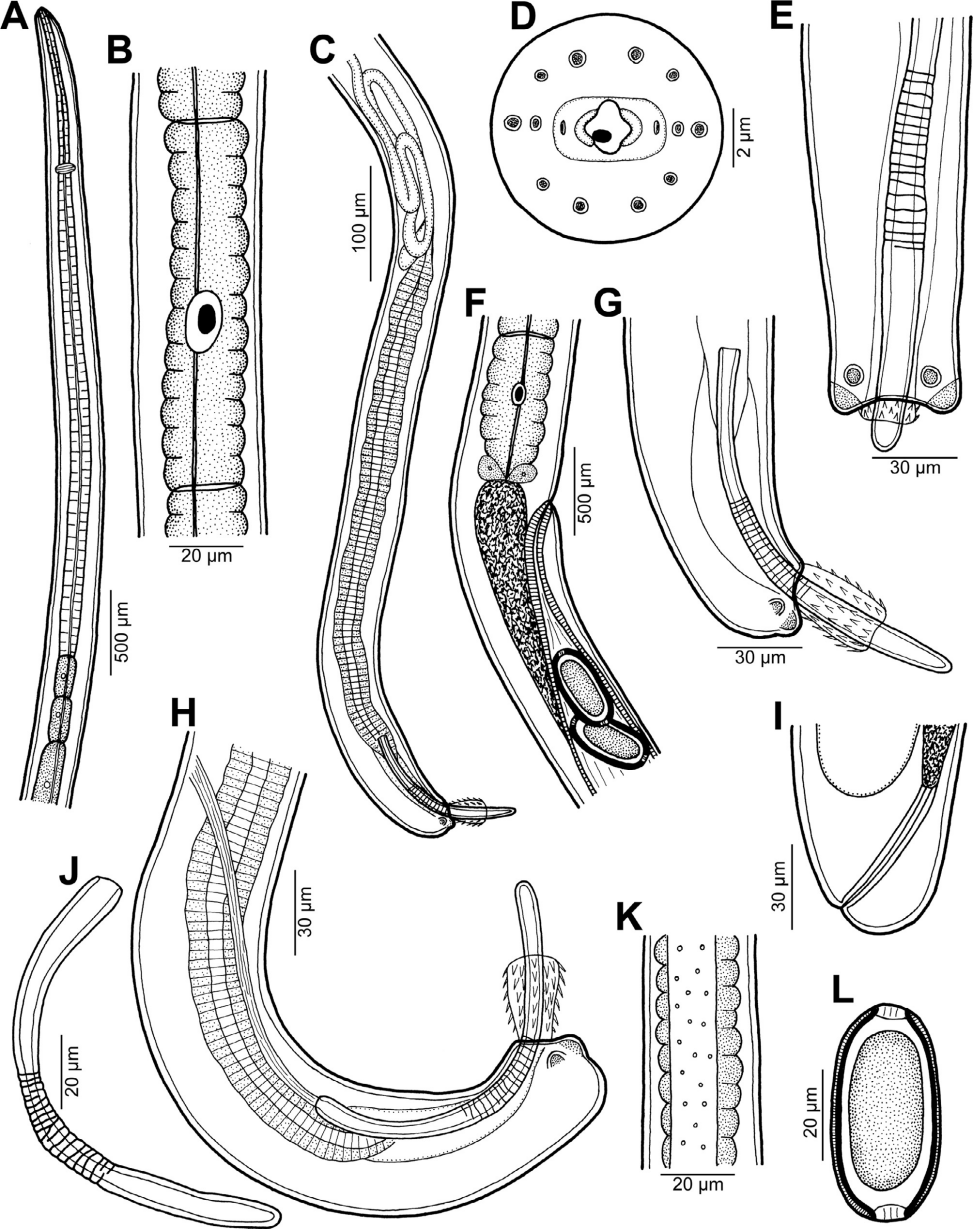
*Capillaria plectropomi* n. sp. from *Plectropomus leopardus*. A: anterior end of male, lateral view. B: stichocyte in middle part of stichosome. C: posterior end of male, lateral view. D: cephalic end of female, apical view. E: caudal end of male, ventral view. F: region of vulva, lateral view. G, H: caudal end of male (different specimens), lateral views. I: tail of female, lateral view. J: spicule, lateral view. K: lateral bacillary band at oesophageal region, lateral view. L: fully developed egg.

**Figure 2.**
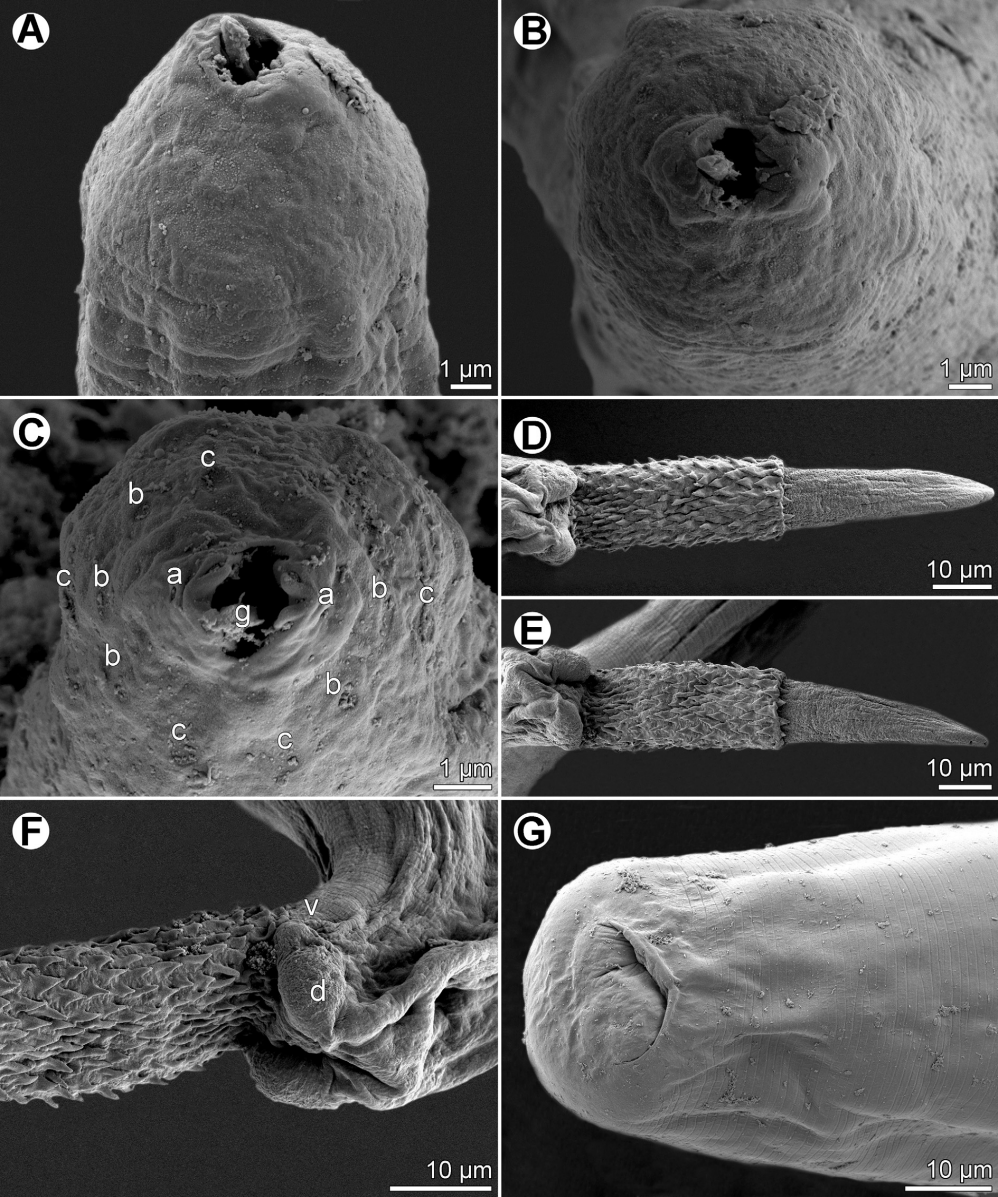
*Capillaria plectropomi* n. sp. from *Plectropomus leopardus*, scanning electron micrographs. A, B: cephalic end, dorsoventral and apical views. C: same, apical view (different specimen). D, E: male caudal end with extruded spicule and spinose spicular sheath, dorsal views (two different specimens). F: caudal end of male, lateral view. G: caudal end of female, ventral view. *Abbreviations*: a, amphid; b, cephalic papilla of inner circle; c, cephalic papilla of outer circle; d, male dorsolateral caudal lobe; g, stylet; v, lateral caudal papilla.

Type host: Leopard coral grouper, *Plectropomus leopardus* (Lacepède) (Serranidae, Perciformes). Small specimen, fork length 280 mm, weight 292 g.

Site of infection: Intestine.

Type locality: Off Baie de Koutio, Nouméa, New Caledonia (collected 6 March 2003).

Prevalence and intensity: 19 specimens found in 1 of 24 fish examined.

Deposition of type specimens: Muséum National d’Histoire Naturelle, Paris (holotype and allotype and paratypes), MNHN JNC243 M and Institute of Parasitology, Biology Centre of the Academy of Sciences of the Czech Republic, České Budějovice (paratypes), Catalogue Number N-1076.

Etymology: The specific name of this nematode relates to the genitive form of the generic name of the host.

### Description

Small, filiform nematodes with finely transversely striated cuticle (Figs. 2A, G). Two inconspicuous lateral bacillary bands extending along body (Fig. 1K). Oral aperture terminal, roughly oval-shaped, oriented dorsoventrally, surrounded by two elevated lips connected in angles of mouth opening. Outer margin of lips rounded, middle of each lip supported from inner side by distinct, small curved mound forming two lobes. Small, finger-shaped stylet with rounded tip and somewhat broader basal part protruding out from buccal cavity (Figs. 1D, 2A–C). Mouth surrounded by 12 cephalic papillae arranged in two circles, each consisting of six papillae, and pair of small lateral amphids located on lips (Figs. 1D, 2A–C). Muscular oesophagus long, narrow. Stichosome consisting of single row of about 40 elongate stichocytes with distinct 6–12 transverse annulae; nuclei of stichocytes large (Fig. 1B). Nerve ring encircling muscular oesophagus at approximately one fourth of its length. Two wing-like pseudocoelomatic glandular cells present at oesophago-intestinal junction (Fig. 1A).

*Male* (six specimens; measurements of holotype in parentheses): Length of body 7.52–10.00 (9.02) mm, maximum width 63–66 (66). Width of lateral bacillary bands at region of posterior end of oesophagus 15–18 (18). Length of entire oesophagus 4.62–5.58 (4.62) mm, representing 51–65% (51%) of body length. Length of muscular oesophagus 330–399 (330), of stichosome 4.29–5.51 (4.29) mm; number of stichocytes about 40 (42). Nerve ring situated 99–108 (102) from anterior extremity. Cloaca 765–1095 (909) long; seminal vesicle tubular, long, forming coils (Figs. 1C, H). Spicular canal well developed, 36–60 (36) long. Spicule well sclerotized, measuring 168–186 (168) in length; proximal end of spicule blunt, 7–12 (9) wide; width of middle part of spicule 9–12 (12); distal end narrowed, rounded, 6 (6) wide. Surface of middle part of spicule with numerous rough transverse grooves (Figs. 1C, E, G, H, J). Spicular sheath spinous, spines about 3 (3) long; length of part of sheath extruded from cloaca 15–33 (33), width 18–21 (21). Posterior end of body rounded, with two distinct, round dorsolateral lobes 6 (6) long, and pair of rather large lateral papillae near base of caudal lobes (Figs. 1C, E, G, H, 2D–F). Cloacal opening subterminal, length of tail 6–12 (6).

*Female* (12 gravid specimens; measurements of allotype in parentheses, those of one nongravid specimen in brackets): Length of body 9.57–14.24 (13.63) [8.43] mm, maximum width 54–96 (96) [57]. Width of lateral bacillary bands at region of posterior end of oesophagus 18–21 (21) [15]. Length of entire oesophagus 4.13–5.15 (4.79) [3.26] mm, representing 35–43% (35%) [39%] of body length. Length of muscular oesophagus 255–363 (330) [326], of stichosome 3.78–4.79 (4.43) [2.94]; number of stichocytes about 40 (about 40) [about 40]. Nerve ring situated 84–111 (105) [84] from anterior extremity. Vulva located 4.13–5.20 (5.15) [3.32] mm from anterior end of body, at 36–43% (38%) [39%] of body length, 0–54 (41) [54] posterior to level of oesophago-intestinal junction. Vulval lips not protruding (Fig. 1F). Vagina short, muscular. Eggs arranged in single file in uterus. Eggs oval, without protruding polar plugs (Figs. 1F, L). Egg wall appearing as two-layered; inner layer hyaline, outer layer with fine superficial net-like sculpture. Eggs including polar plugs 60–66 × 27 (60 × 27) [-], thickness of egg wall 2–3 (2); polar plugs 3–4 (4) long and 6 (6) wide. Content of fully developed eggs uncleaved. Caudal end rounded, anus subterminal (Figs. 1I, 2G); tail 12–15 (12) [12] long. Rectum formed by hyaline tube 60–66 (66) [45] long (Fig. 1I).

## Discussion

According to Moravec [9], capillariid nematodes are represented by species in 22 genera, of which nine genera comprise parasites of freshwater, marine and brackish-water teleost fishes and elasmobranchs. In having the stichosome consisting of a single row of stichocytes and males possessing two caudal lobes without a membranous bursa, the spicule with numerous rough transverse grooves on its surface, the spiny spicular sheath and the absence of lateral caudal alae, the present material belongs to *Capillaria* Zeder, 1800 s.s., as diagnosed by Moravec [7].

*Capillaria* includes parasites of all classes of vertebrates except reptiles. Moravec [8] established four subgenera for *Capillaria* spp. from fishes. The general morphology of *C*. *plectropomi* n. sp., in particular the structure of the male caudal end, a heavily sclerotized spicule and the absence of a vulval appendage, shows that this species belongs to the subgenus *Neocapillaria* Moravec, 1987 [8, 9]. To date, this subgenus includes seven species: *C*. *acanthopagri* Moravec, Nagasawa & Madinabeitia, 2010, *C*. *carioca* Freitas & Lent, 1935, *C*. *cooperi* Johnston & Mawson, 1945, *C*. *hakofugu* Araki & Machida, 1991, *C*. *navonae* Timi, Rossin & Lanfranchi, 2006 and *C*. *wickinsi* Ogden, 1965 from marine and brackish-water tetraodontiform, perciform or pleuronectiform fishes in the Atlantic, Indian and Pacific Oceans, and the type species *C*. *pterophylli* Heinze, 1933 from freshwater Neotropical cichlids [12].

The new species differs from all congeneric species belonging to *Neocapillaria* from marine fishes in the length of spicule, which is distinctly shorter in *C*. *cooperi* (120–150 μm vs. 168–186 μm) and *C*. *hakofugu* (110–150 μm) or distinctly longer in *C*. *acanthopagri* (204–285 μm), *C*. *carioca* (438–513 μm), *C*. *navonae* (210–260 μm) and *C*. *wickinsi* (534–660 μm). Moreover, in contrast to the new species, the spicules of *C*. *acanthopagri*, *C*. *carioca* and *C*. *wickinsi* have transverse grooves extending along almost their entire lengths (vs. restricted to the middle part of spicule). The spicules of *C*. *cooperi* and *C*. *pterophylli* are conical and markedly broad at their posterior parts, and a pair of papillae located anterior to caudal lobes is absent in *C*. *hakofugu*.

Whereas the hosts of *C*. *carioca*, *C*. *hakofugu*, *C*. *navonae* and *C*. *wickinsi* belong to other fish orders (Anguilliformes, Pleuronectiformes or Tetraodontiformes), those of *C*. *acanthopagri*, *C*. *cooperi* and *C*. *pterophylli* belong to Perciformes as does that of the new species. However, *C*. *plectropomi* n. sp. differs from the three last-named species in the host family, i.e., Serranidae vs. Sparidae, Callionymidae and Cichlidae, respectively. *Capillaria plectropomi* n. sp. is the first nominal species of this genus described from a representative of the Serranidae. Capillariids in serranids are obviously rare, as demonstrated by the scarcity of our findings in many grouper species and specimens examined in New Caledonia [6]. Smales [13] reported *Capillaria* sp. from *Epinephelus ongus* (Bloch) and *E*. *tauvina* (Forsskål) (Serranidae) off the Keppel Islands, Queensland, Australia and the possibility cannot be excluded that they are *C*. *plectropomi*.

The use of SEM has shown some details in the structure of the cephalic end in *C*. *plectropomi* that are not usually visible in capillariids under the light microscope. It is apparent from this study that the structure of lips, shape of the oral aperture and the number and arrangement of the cephalic papillae of this species are very similar to those described by Baruš et al. [2] in *Capillaria anatis* (Schrank, 1790), by Moravec [10] in *Paracapillaria philippinensis* (Chitwood, Velasquez & Salazar, 1968), by González-Solís et al. [5] in *Capillostrongyloides morae* González-Solís, Carrassón & Pérez-del-Olmo, 2014 or as observed in some species of *Eucoleus* Dujardin, 1845 (unpublished).

The present study also shows the presence of a functional stylet in *C*. *plectropomi*. As mentioned by Moravec [10], the stylet is present in the first-stage larva in all trichinelloids [1], but it is customary to assume that it is absent in the adult stage. Wright [14] observed the stylet in the dorsal portion of the buccal cavity in the adult capillariid *Calodium hepaticum* (Bancroft, 1893), mentioning that it can probably not be projected out of the mouth as in the conspecific first-stage larva. However, the presence of a functional stylet was clearly demonstrated in adult *Paracapillaria philippinensis* [3, 10], *Capillaria anatis* [2] and *Capillostrongyloides morae* [5]. Its finding also in *Capillaria plectropomi* suggests that the functional stylet may be present in all adult capillariids.
